# A case of mid‐ureteral stricture with ipsilateral atrophic kidney in a young adult

**DOI:** 10.1002/iju5.12620

**Published:** 2023-08-28

**Authors:** Arisa Machida, Masakazu Abe, Shuhei Ishii, Kie Sekiguchi, Kenta Takahashi, Ei Shiomi, Shigekatsu Maekawa, Yoichiro Kato, Noriyuki Uesugi, Wataru Obara

**Affiliations:** ^1^ Department of Urology Iwate Medical University School of Medicine Yahaba Japan; ^2^ Department of Pathology Iwate Medical University School of Medicine Yahaba Japan

**Keywords:** diagnosis, kidney, nephroureterectomy, stricture, young adult

## Abstract

**Introduction:**

Most congenital ureteral strictures occur at the ureteropelvic or ureterovesical junction in children. Mid‐ureteral stricture is very rare and can cause congenital hydronephrosis. Only a few studies have reported on coexisting mid‐ureteral stricture with ipsilateral atrophic kidney in young adults.

**Case presentation:**

A 16‐year‐old girl presented with repeated urinary tract infection. Computed tomography revealed a right atrophic kidney and hydroureter. Retrograde pyelography showed a mid‐ureteral stricture. Laparoscopic nephroureterectomy was performed, and histological examination revealed mid‐ureteral stricture with hyperplasia of the fibrous connective tissue and an atrophic kidney.

**Conclusion:**

Mid‐ureteral stricture in a young adult is extremely rare. Appropriate imaging studies including retrograde pyelography are necessary for accurate diagnosis of mid‐ureteral stricture.

**Abbreviations & Acronyms**
CT: computed tomography
CTU: CT urography
DTPA: diethylenetriamine pentaacetic acid
EM: elastica‐masson staining
HE: hematoxylin–eosin
MRI: magnetic resonance imaging
MRU: magnetic resonance urography
RP: retrograde pyelography
UPJO: ureteropelvic junction obstruction
UTI: urinary tract infection
UVJO: ureterovesical junction obstruction
VCG: voiding cystography
VUR: vesicoureteral reflux

**Keynote message**

We present a case of mid‐ureteral stricture with an ipsilateral atrophic kidney in a young adult. Appropriate imaging examinations are necessary for accurate diagnosis of mid‐ureteral stricture.

## Introduction

Mid‐ureteral stricture is very rare and can cause congenital hydronephrosis. We report a case of mid‐ureteral stricture with an ipsilateral atrophic kidney, which required laparoscopic nephroureterectomy, in a young adult.

## Case presentation

**Fig. 1:**
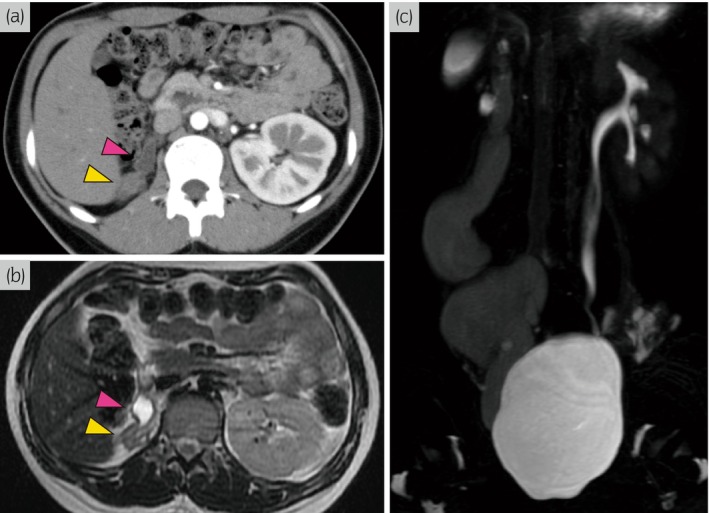
Imaging findings. (a) Enhanced CT image shows the right atrophic kidney (yellow arrow) and hydroureter (pink arrow). (b) MRI shows right atrophic kidney (yellow arrow) and hydroureter (pink arrow). (c) MRU reveals marked hydroureter on the right side.

**Fig. 2:**
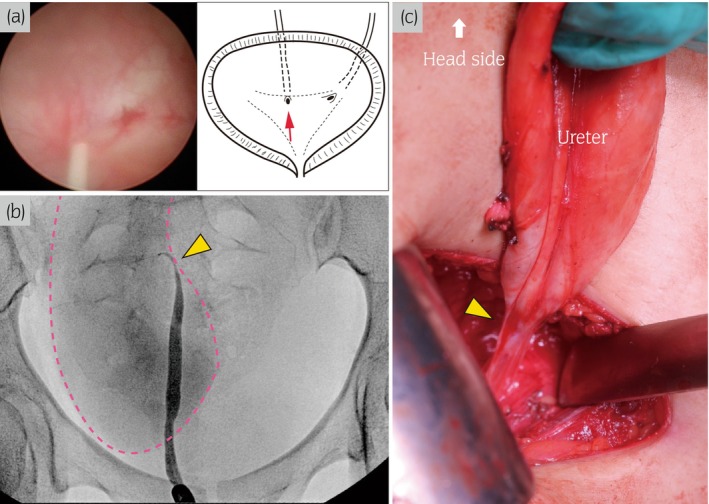
RP and intraoperative findings. (a) Cystoscopy shows that the right ureteral orifice is displaced to the midline of the bladder trigone. The illustration shows the location of the right orifice (red arrow). (b) RP reveals mid‐ureter stricture (yellow arrow) and dilation of the upper ureter (pink dotted line). (c) Intraoperative image shows the mid‐ureter stricture part without abnormal blood vessel crossing (yellow arrow).

**Fig. 3:**
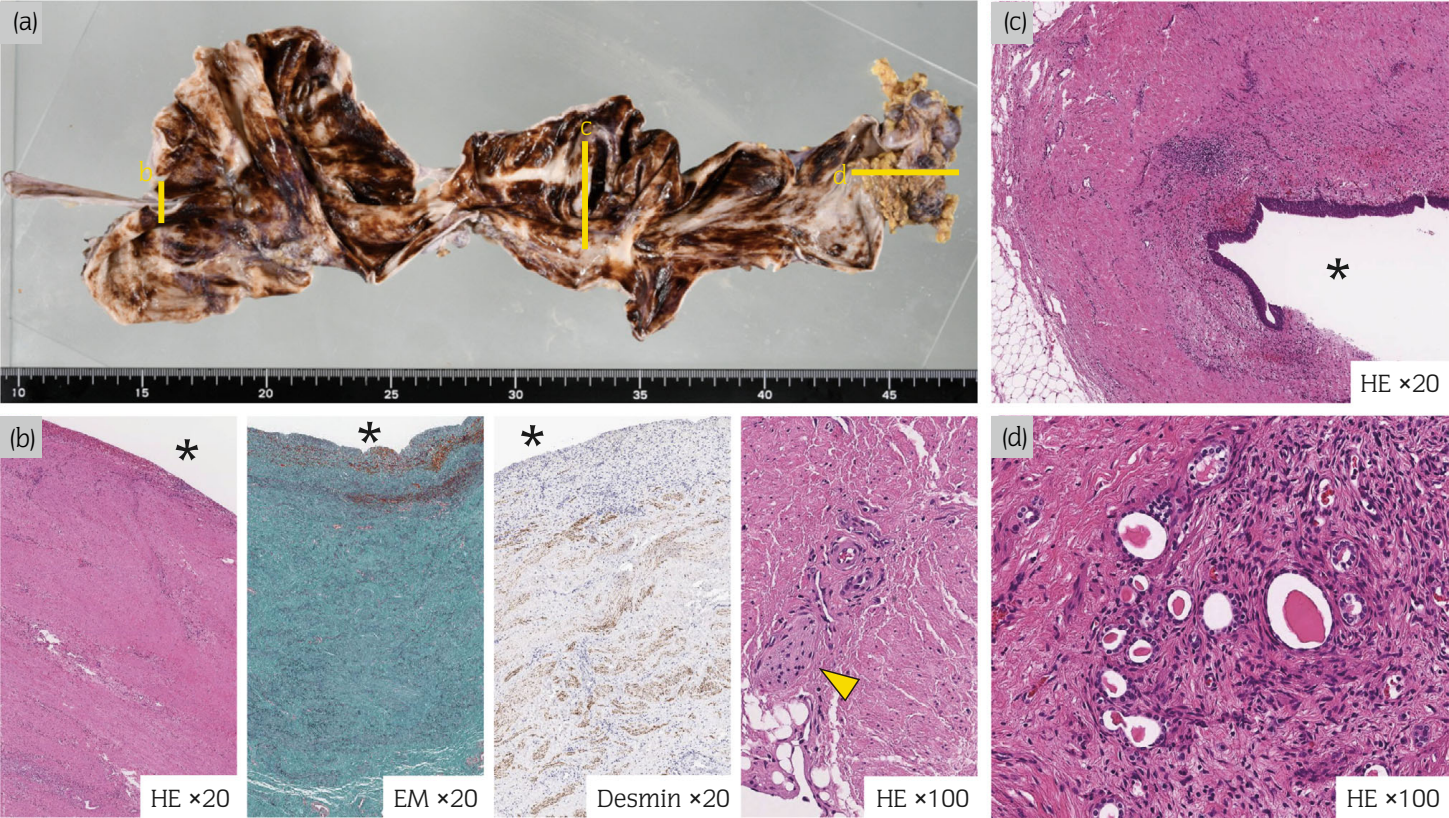
Pathological examination findings. (a) Macroscopic findings show an atrophic kidney and extreme ureter dilation. Line‐b is the stricture site, line‐c is the dilatation site, and line‐d is the atrophic kidney. (b) Pathological examination findings of the ureteral stricture site. The asterisk indicates the luminal side. Pathological examination findings reveal that the ureteral stricture is thickened due to hyperplasia of fibrous connective tissue (HE staining ×20, EM × 20). The smooth muscle tissue remains (desmin staining ×20), and yellow arrows indicate that nerve fibers also remain (HE ×100). (c) Pathological examination findings at the ureteral dilatation reveal that the muscular layer is maintained, with mild inflammatory cell infiltration (HE ×20). (d) Pathological examination findings of the kidney reveal that the renal tubules are almost completely atrophied, and glomeruli are not identified (HE ×100).

A 16‐year‐old girl was referred to our institute due to repeated UTI. She had mild right hydronephrosis from birth without VUR. Thereafter, she was observed regularly and followed up by pediatrics, who also recognized right atrophic kidney gradually. However, a detailed urinary tract examination had not been performed. Physical examination at our department revealed no obvious malformations in other organs. Laboratory findings were within normal limits (serum creatinine, 0.63 mg/dL), except for mild bacteriuria. Enhanced CT and MRI showed an atrophied kidney and dilated ureter on the right side (Fig. 1a,b). In particular, the right ureter appeared as a giant ureter on the sagittal section of MRU (Fig. 1c). Technetium‐99m DTPA renal scintigraphy showed no accumulation in the right kidney. VCG showed no VUR. The right ureteral orifice was displaced in the midline of the bladder trigone, and no abnormal morphological findings of the orifice were found on cystoscopy (Fig. 2a). RP showed that the length of the mid‐ureter stricture was about 10 mm. Although the urinary tract above the stricture was markedly expanded, the lower ureter below the stricture was not dilated (Fig. 2b). We performed a right nephroureterectomy with a diagnosis of mid‐ureter stricture with an ipsilateral non‐functional kidney. We tried to perform reduced‐port laparoscopic surgery for cosmetic purposes. A GelPOINT access platform was placed between the tip of the 12th rib and the iliac crest. The renal artery and vein were ligated by retroperitoneal approach. As the ureter was markedly adherent to the surrounding tissue at the stricture, we added a 5‐cm incision in the right lower abdomen and extracted the right kidney and ureter (Fig. 2c). No abnormal blood vessels were found around the stricture site. Macroscopic findings revealed mid‐ureter stricture and atrophic kidney (Fig. 3a). The lower ureter below the stricture did not dilate, similar to the findings on preoperative RP. Pathological examination findings confirmed that the mid‐ureteral stricture was thickened due to hyperplasia of fibrous connective tissue and that the smooth muscle tissue and nerve fibers remained in place (Fig. 3b). Moreover, the muscular layer was maintained in ureteral dilatation, and mild inflammatory cell infiltration was observed (Fig. 3c). The renal tubules were almost completely atrophied, and glomeruli could not be identified. The patient was discharged without any notable perioperative events. Currently, 3 years have passed without a relapse of UTI.

## Discussion

Most upper urinary tract obstruction lesions are commonly UPJO and UVJO, and stricture in the middle ureter is extremely rare. Congenital mid‐ureter stricture is represented by a narrowing that occurs between the UPJ and UVJ. Campbell reported only 4% had mid‐ureter stricture in an autopsy of a sequence of 72 children who had ureteral stricture.^1^ Most recently, Meng et al. reported that middle ureteral stricture accounts for only 1.6% of all ureteral strictures.^2^

Mid‐ureteral stricture occurs at the level of the bifurcation of the common iliac vessels. Previous literature has shown that the ureteral lumen is shrunken by around 60% at the stricture site, resulting in a significant obstruction to urine delivery.^3^ Meng et al. reported that children with mid‐ureteral stricture had relatively mild hydronephrosis and delayed onset of symptoms.^2^ Few studies have reported on mid‐ureteral stricture diagnosed at 15 and 20 years of age.^4^, ^5^ Hawang et al. reported that renal ultrasound and radionuclide renography alone do not suffice to identify the site of stricture, and that preoperative RP should be performed to identify the stricture site unless the distal ureter is well confirmed by other tests.^6^ If the distal ureter is not clearly visible or the diagnosis is unclear, MRU, CTU, or RP is performed to confirm the diagnosis. In our case, the hydronephrosis in childhood was mild, and the patient was followed up without a detailed examination of the ureter. We considered that mid‐ureter stricture causes repeated UTIs and severely atrophied kidney, resulting in renal function abolishment.

The pathogenesis of congenital mid‐ureter stricture is unclear, and many theories attribute it to abnormal embryonic development, including abnormal fetal vessel compression, intrauterine inflammation, incomplete ureteral recanalization, ischemia due to abnormal branches of blood vessels, and localized developmental arrest.^3^, ^6^, ^7^, ^8^, ^9^ Previous review reported that congenital mid‐ureter stricture is often associated with urological anomalies such as contralateral renal agenesis or atrophy, VUR, UPJO, solitary kidney, and ectopic ureteral opening. In our case, shifting of the ureteral orifice to the midline was observed.

In pathology, ureteral stricture is defined as a mechanical obstruction due to structural abnormalities in the wall. Two pathogenesis of ureteral stricture exist: ureteral valve and true ureteral stricture. The ureteral valve is a transverse fold of ureteral mucosa with anatomically proven.^5^ In our case, there was obvious luminal narrowing, but no valve was detected. Studies of the ultrastructure of ureteral stricture revealed that the stenotic ureter exhibits only quantitative changes in its composition. These changes included lumen shrinkage and relative or absolute loss of smooth muscle with a normal, altered, or disorganized arrangement, with or without connective tissue changes.^10^, ^11^, ^12^ In our case, pathological examination revealed that the area of ureteral stricture was thickened due to hyperplasia of fibrous connective tissue and that the smooth muscle tissue and nerve fibers remained. The stricture site showed a low level of chronic inflammatory cell infiltration, which is consistent with findings reported in the literature.

General treatments for mid‐ureteral stricture include excision of impaired passages and end‐to‐end anastomosis of the ureter. In adult cases, endoscopic procedures such as antegrade or retrograde endoureterotomy may be performed. In pediatric cases, endoscopic incision or dilation of the stenosis may be considered, but the success rate is lower than that of resection and anastomosis. Recently, treatment for ureteral stricture using laparoscopic and robotic technology is also available. Compared with open surgery, minimally invasive surgery has the advantages of less postoperative pain, shorter hospital stay, and less scarring. In the future, more patients will be able to undergo treatment with laparoscopic and robotic surgery.

## Conclusion

We report a case of a young adult with mid‐ureteral stricture with ipsilateral atrophic kidney, which required nephroureterectomy. A detailed image examination, including RP, is necessary for definitive diagnosis of mid‐ureteral stricture.

## Author contributions

Arisa Machida: Conceptualization; writing – original draft. Masakazu Abe: Conceptualization; writing – review and editing. Shuhei Ishii: Data curation. Kie Sekiguchi: Data curation. Kenta Takahashi: Data curation. Ei Shiomi: Conceptualization. Shigekatsu Maekawa: Supervision. Yoichiro Kato: Conceptualization; supervision; writing – review and editing. Noriyuki Uesugi: Visualization. Wataru Obara: Supervision; writing – review and editing.

## Conflict of interest

The authors declare no conflict of interest.

## Approval of the research protocol by an Institutional Reviewer Board

Not Applicable.

## Informed consent

Informed consent was obtained from the patient for the publication of this case report and the accompanying images.

## Registry and the Registration No. of the study/trial

Not Applicable.
